# Research Progress on Long Noncoding RNAs and N6-Methyladenosine in Hepatocellular Carcinoma

**DOI:** 10.3389/fonc.2022.907399

**Published:** 2022-07-22

**Authors:** Wenjie Zhang, Wenlong Wu, Qiang Meng, Long Yang, Juzheng Yuan, Zelin Tian, Rui Ding, Xuan Zhang, Jianlin Wang, Kaishan Tao

**Affiliations:** ^1^ Chinese Education Ministry’s Key Laboratory of Western Resources and Modern Biotechnology, Key Laboratory of Biotechnology Shaanxi Province, College of Life Sciences, Northwest University, Xi’an, China; ^2^ Department of Hepatobiliary Surgery, Xijing Hospital, The Fourth Military Medical University, Xi’an, China; ^3^ Xi’an Medical University, Xi’an, China

**Keywords:** hepatocellular carcinoma, long noncoding RNAs, epigenetic modification, methylation modification, N6-methyladenosine

## Abstract

N6-methyladenosine (m6A) is an epigenetic modification that widely exists in long noncoding RNAs (lncRNAs) and is involved in the regulation of oncogenes or tumor suppressor genes that form complex enzymes to affect the occurrence of tumors. The abnormal modification of m6A methylation can alter the overall m6A level and thus contribute to the malignant biological behaviors of hepatocellular carcinoma (HCC). LncRNAs related to m6A methylation are involved in lipogenesis, the proliferation, migration and invasion of HCC cells, the stemness of tumor cells and sorafenib resistance. In this review, we systematically elaborated the occurrence mechanism of lncRNA and m6A methylation modification in HCC and the effect of m6A methylation modification of lncRNA on the occurrence of HCC, suggesting that the combination of m6A methylation modification and lncRNA will be more meaningful as molecular markers or prognostic markers. It is helpful to provide further ideas for exploring the pathogenesis of HCC and identifying new targets for HCC treatment and diagnosis and achieve precise individual treatment of liver cancer.

## 1 Introduction

Liver cancer is the third most common cause of cancer death in the world, after lung cancer and colorectal cancer, with 905,677 new cases and 830,180 deaths each year, accounting for 8.3% of the total number of deaths from HCC (1). HCC is the most common pathological type of primary liver cancer, accounting for 75%-85% of cases. China is a high-risk area for HCC, with 50% of the new cases diagnosed annually worldwide, showing an obvious upward trend (2). In recent years, the overall prognosis of HCC is still poor, with a 5-year survival rate of only 15-38%, despite the improved survival of patients with multidisciplinary comprehensive treatment and personalized treatment programs (3–5).

In recent years, studies on the pathogenesis of HCC have focused on oncogenes, tumor suppressor genes, epigenetic modification and noncoding RNA (ncRNA). With the development of high-throughput sequencing technology, researchers have found that m6A modification sites are widely present on messenger RNA (mRNA) and lncRNA (6, 7). m6A modification affects gene expression by regulating mRNA and lncRNA and abnormal expression of lncRNA levels and m6A methylase complexes associated with various human cancers, including HCC (8, 9). These findings suggest that these differentially expressed genes may provide guidance for the study of the pathogenesis of HCC and provide a new target for the diagnosis or treatment of HCC. Therefore, this paper reviews the mechanism of m6A modification and the lncRNAs involved in the pathogenesis and development of HCC. A detailed study of the distribution and function of lncRNAs and chemical modifications as well as their association with related proteins will contribute to a comprehensive understanding of the multilevel gene expression regulation mechanism in mammalian cells. The potential role of m6A modification at the level of lncRNA may provide additional mechanisms for transcriptome gene regulation during body development. The study of lncRNA and m6A can further explore tumor markers and contribute to the detection and diagnosis of HCC. The combination of m6A methylation modification of lncRNA with clinical drug therapy and emerging immunotherapy provides new opportunities for early diagnosis, effective treatment and even disease prognosis of cancer.

## 2 m6A Methylation Modification

### 2.1 Overview of m6A

**Figure 1:**
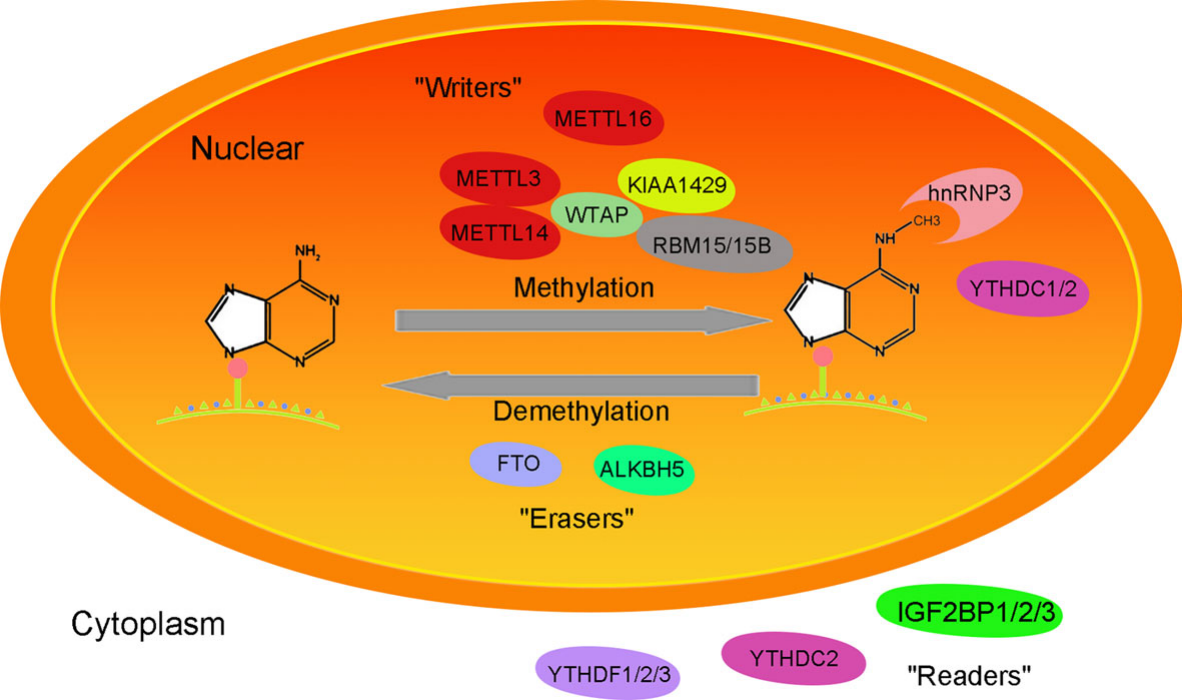
Mechanism of the function of the m6A methylation-related enzyme complex.

The m6A methylation modification refers to methylation of the N atom at the sixth position of adenine (A) and was first discovered in 1974. m6A methylation sites mostly appeared on the common sequence RRACH (R = A/G; H for A/C/U), and there were more 3’ -UTR (3’-untranslated regions), CDS (coding sequence), intron and stop codon regions and fewer 5’ UTR regions (10, 11) (**Figure 1**).

### 2.2 m6A Methylation-Related Enzymes

#### 2.2.1 Methyltransferase Complex (Writers)

Methyltransferase complexes include METTL3 (methyltransferase-like 3), METTL14 (methyltransferase-like 14), WTAP (Wilms tumor 1-associated Protein), KIAA1429 (also called VIRMA, vir-like m6A methyltransferase associated), RBM15/15B (RNA-binding motif protein 15/15B), METTL16 (methyltransferase-like 16) and so on. Its function is to add methyl groups on the S-adenosyl methionine transferase to the target RNA. METTL3, as the catalytic core of methylase, has methylase activity (12). As an RNA binding domain, METTL14 can enhance methylase activity by binding METTL3 (13). WTAP stabilizes the METTL3/METTL14 complex, helps METTL3/14 locate nuclear spots, and maintains the catalytic activity of m6A methyltransferase *in vivo*(14). RBM15/RBM15B recruits METTL3-related methylation complexes to act on specific methylation sites in a WTAP-dependent manner. METTL16 acts as a methyltransferase, catalyzing m6A methylation of U6 snRNA and ncRNA (15).

#### 2.2.2 Demethylases (Erasers)

Fat mass and obesity-associated protein (FTO) and a-ketoglutarate-dependent dioxygenase alkB homolog 5 (ALKBH5) belong to the α-ketoglutarate-dependent dioxygenase family and remove m6A methylation in an Fe2+- and α-ketoglutarate-dependent manner (16). FTO plays a key role in regulating m6A modification of mRNA transcription and is an important m6A demethylase associated with metabolic disorders such as diabetes and obesity (17). In addition to m6A demethylation, FTO also acts on m6Am of mRNA/snRNA (small nuclear RNA) and m1A of tRNA (18, 19). ALKBH5, a member of the alkB family, demethylates m6A on single-stranded RNA/DNA and can directly remove methyl groups from m6A-methylated adenosine (20). ALKBH5 is expressed differently in different tissues, with high expression in the testis but low expression in the heart and brain. ALKBH5 plays a role in nuclear RNA output and metabolism, gene expression and mouse fertility (21).

#### 2.2.3 m6A Methyl Recognition Protein (Readers)

The methylated molecules combine with the m6A methyl recognition protein “readers” (YTHDF1/2/3, YTHDC1/2, HNRNPA2B1, HNRNPC and HNRNPG, IGF2BP1/2/3) to perform their epigenetic modification (22, 23). It affects alternative splicing, output, translation and stability of organisms. IGF2BP1/2/3 mainly plays a role in maintaining RNA stability (12). YTHDC1 exists in the nucleus, regulates the selective splicing of RNA, maintains the stability of RNA, and has a certain nuclear output function (22). YTHDF1/YTHDF3/YTHDC2 can interact with promoters and participate in RNA translation, while YTHDF2 regulates RNA degradation (23).

Abnormal expression of m6A methylation regulators can change the overall intracellular m6A level, affect RNA splicing, stabilization, output, and transcription, and affect the expression of downstream target genes by regulating the binding of RNA binding proteins (RBPs) or miRNAs (22–24). Furthermore, it can regulate the cell cycle, cell differentiation and development, and carcinogenesis.

### 2.3 Abnormal Expression of m6A Affects HCC Processes

#### 2.3.1 Impact of the Abnormal Expression of “Writers” in HCC

Studies have shown that METTL3 expression in HCC is significantly higher than that in normal tissue, and METTL3 plays an important role in HCC. Overexpression of METTL3 can increase the m6A modification of SOCS2 (suppressor of cytokine signaling 2) mRNA, reducing the stability of the downstream target SOCS2 mRNA and inhibiting the expression of SOCS2, thus promoting the proliferation and migration of HCC cells (25). In addition, METTL3 is associated with a poor prognosis in HCC patients (26). It has been reported that METTL14 is expressed at low levels in HCC and inhibits HCC metastasis by interacting with DGCR8 (DiGeorge syndrome critical region gene 8) and promoting the maturation of pri-miRNA126. It can thus be used as one of the predictors of liver cancer recurrence and metastasis (27).

#### 2.3.2 Impact of the Abnormal Expression of “Erasers” in HCC

The expression of FTO has been found to be upregulated in HCC tissues and cells, and high expression of FTO is associated with a poor prognosis in HCC patients (28). FTO knockdown obstructs the transition from G0 phase to G1 phase and inhibits the growth of tumor cells (17). Highly expressed FTO acts as a m6A demethylase to induce the demethylation of PKM2 (pyruvate kinase M2) mRNA and promote the production of translation products, thus affecting the occurrence of HCC (29). As a tumor suppressor, ALKBH5 is high-expressed in HCC tissues and cells. ALKBH5-mediated m6A demethylation can lead to posttranscriptional inhibition of LYPD 1 (LY6/PLAUR domain containing 1). LYPD1 can then be recognized and stabilized by the m6A effector IGF2BP1, exerting its oncogene function to promote the proliferation and invasion of HCC (30).

#### 2.3.3 Impact of the Abnormal Expression of “Readers” in HCC

Notably, both the oncogene YTHDF1 and the suppressor factor YTHDF2 are involved in the occurrence and development of HCC. YTHDF1 is significantly overexpressed in HCC and is closely related to the pathological stage and survival rate of patients (28). Studies have found that c-Myc is the upstream gene regulating YTHDF1, and it is speculated that the potential target gene regulating YTHDF1 protein may be related to the tumor cell cycle, the degradation of various amino acids and lipid metabolism, and their abnormal expression in cells may lead to the occurrence of HCC (31). IGF2BPs are highly expressed in hepatocellular carcinoma cells and are associated with poor prognosis. IGF2BP1 promotes the expression of serum response factor (SRF) and its target genes in a m6A-dependent manner, enhances the expression of tumor genes and promotes the proliferation, migration and invasion of HCC cells (32).

## 3 LncRNA

### 3.1 Overview of LncRNAs

In the past few years, with the rapid development of second-generation sequencing technology, it has been determined that most of the genome is transcribed into RNA, while only 1-2% of the RNA with protein coding ability is RNA, most of which is ncRNA (33). According to the number of ncRNA nucleotides, ncRNAs with less than 200 nucleotides are called short noncoding RNAs (including microRNAs, circRNAs, siRNAs and piRNAs), and ncRNAs with a length greater than 200 nucleotides are called lncRNAs (34).LncRNAs were classified based on the relationship between the genome and protein-coding genes: a) justice lncRNAs, which overlap with one or more exons of coding genes; b) antisense lncRNAs, which are partially or completely complementary to the transcriptional products on the opposite chain; c) intronic lncRNAs, which are derived from introns of genes; d) bidirectional transcripts, which share the same promoter with protein-coding genes but are transcribed in opposite directions; and e) intergenic lncRNAs, which are independently transcribed by sequences located between protein-coding genes (34–36) (**Table 1**).

**Table 1:** LncRNA classification.

Classification	Based on the genome and the relationship between lncRNA and protein-coding genes
Justice LncRNA	Overlaps with one or more exons of the coding gene
Antisense LncRNA	Partially or completely complementary to transcripts on opposite chains
Intronic LncRNA	Produced by genetic introns
Bidirectional transcript	It has the same promoter as the protein-coding gene, but the transcription direction is opposite
Intergenic LncRNA	Independently transcribed between protein-coding genes

LncRNAs exist in different subcellular structures, including the cell membrane, cytoplasm and nucleus, and their functions and regulatory mechanisms are closely related to their localization in cancer cells. In the nucleus, lncRNAs can act as scaffolders to recruit regulatory proteins and interact with mRNAs or as competing endogenous RNAs (ceRNAs) to regulate mRNA translation and stability (35). Moreover, lncRNAs may be involved in maintaining the stability of m6A-related proteins. LncRNAs are diverse and have complex functional mechanisms. They play vital regulatory roles in gene expression, species evolution, embryonic development, the metabolism of substances and tumorigenesis and are important branches and hotspots of transcriptional regulation (37). Studies have shown that lncRNAs are abnormally expressed in a variety of cancers and play a role as key tumor promoters or inhibitors, affecting cell growth, apoptosis and metastasis (36).

### 3.2 The Role of LncRNAs in HCC

It has been confirmed that lncRNAs can perform their functions through epigenetic modification, transcription, splicing and degradation and can interact with downstream target molecules (mRNA, microRNA and protein) directly or with the help of transcription factors to regulate the expression of oncogenes or tumor suppressor genes and activate intracellular signaling pathways (38). They can affect cell proliferation, differentiation, metastasis and apoptosis, further affecting the occurrence and development of HCC (39).

LncRNA-CDKN2B (cyclin-dependent kinase inhibitor 2B) expression is upregulated and changes the chromatin structure by recruiting chromatin modification factors to regulate the expression of target genes, ultimately promoting the proliferation of HCC cells (40). LncRNA-HULC (highly upregulated in liver cancer) can interact with the promoter of protein-coding genes to promote the expression of the autophagy protein P62 and activate the AKT-PI3K-mTOR pathway (41, 42). At the same time, it synergizes with lncRNA-MALAT1 (metastasis-associated lung adenocarcinoma transcript 1) to upregulate the transcriptional expression of telomerase repeat binding protein 2, which jointly enhances the immortalization of this protein and reduces the methylation of the telomerase RNA promoter region (43). LncRNAs can promote transcription in this region, thus maintaining telomere length and influencing the occurrence of HCC.

There are miRNA-binding sites on lncRNAs, which can act as ceRNAs to regulate the expression of downstream target genes and corresponding signaling pathways through competitive binding miRNAs, thus affecting the proliferation, cycle and metastasis of HCC (39). LncRNA-GAS5 (growth arrest-specific transcript 5) is overexpressed in liver cancer tissues and cells and negatively regulates miR-21, indirectly affecting the expression of the downstream target gene PTEN and thus regulating the proliferation of HCC. Meanwhile, the high expression of lncRNA-GAS5 was correlated with tumor stage (TNM), overall survival (OS), disease-free survival (DFS) and metastasis, suggesting that GAS5 may be a potential diagnostic and prognostic biomarker in HCC (44). LncRNA-H19 was significantly correlated with vascular infiltration and promoted the expression of alpha-fetoprotein (AFP) (45). LncRNA-CCAT1 (colon cancer-associated transcript 1) may play a role as a proto-oncogene in HCC and is upregulated in cancer tissues. The high level of its expression is correlated with tumor size, vascular invasion and serum AFP level and may be related to the prognosis of HCC patients (46). LncRNA-TUC338 and lncRNA-VLDLR are highly expressed in HCC cells and tissues and are associated with sorafenib resistance, suggesting a role for lncRNAs in clinical treatment (47, 48).

## 4 Current Research Progress on LncRNA and m6A Methylation in HCC

**Figure 2:**
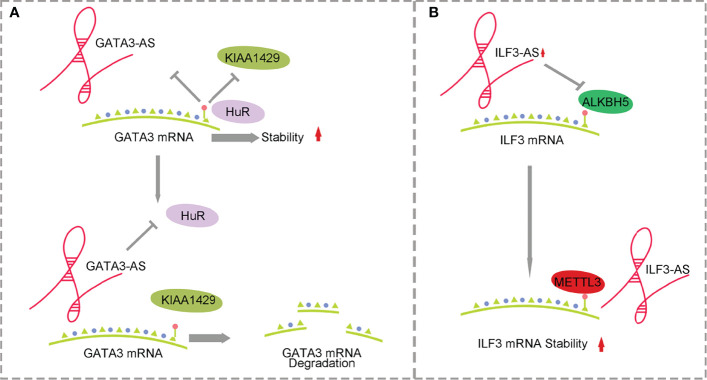
Mechanism of antisense lncRNA and m6A methylation affecting HCC. **(A)** GATA3-AS with the help of methylase KIAA1429 promotes the degradation of GATA3 mRNA. **(B)** ILF3-AS with the help of METTL3 improves ILF3 mRNA stability.

**Figure 3:**
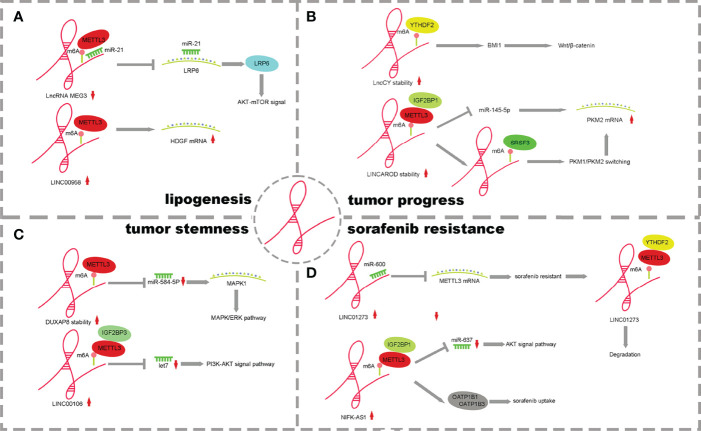
The mechanism of lncRNA m6A regulatory factors and their influence. **(A)** m6A of lncRNAs promote lipogenesis. **(B)** m6A of lncRNAs effect HCC Processes. **(C)** m6A of lncRNAs enhance tumor stemness. **(D)** m6A of lncRNAs influence drug resistance on tumor cells.

Early studies on m6A methylation focused on mRNA. With the rapid development of high-throughput sequencing, lncRNA CHIPS and MeRIP technology, researchers have found that lncRNAs have important value in tumors. Studies on the m6A methylation of lncRNAs have become the focus of attention (24). Dysregulation of lncRNA and m6A regulator factors in cancer has been described in a large number of studies in gastric cancer, non-small-cell lung cancer, and prostate cancer (49–51). However, there are few studies on how lncRNA and m6A methylation modification regulate the pathogenesis of HCC. An increasing number of reports have proven that lncRNAs and m6A regulatory factors can be used as biomarkers or therapeutic targets for liver cancer (52). This suggests that it is of great significance to explore the m6A methylation of lncRNAs in the pathogenesis of HCC. Currently, the effects of lncRNAs and m6A on the occurrence and progression of HCC are shown in **Figures 2**, **3** and **Table 2**.

**Table 2:** The mechanism of m6A regulatory factors and their influence on HCC.

LncRNA	Expression	m6A regulatoryfactor	Function	Mechanism	Refs
MEG3	Down	METTL3	Inhibits the proliferation, invasion and migration of HCC	Through the ceRNA mechanism, the expression of miR-544b is promoted, and the target gene BTG2 is downregulated	(53, 54)
LINC00958	Up	METTL3	Promotes lipogenesis and HCC processes	METTL3 increased the stability of LINC00958, inhibited the expression of miR-3619-5p and increased the expression of HDGF	(55)
LNCAROD	Up	METTL3 IGF2BP3	Promotes HCC progression and chemotherapy resistance	LNCAROD binds to SRSF3 to induce PKM1-PKM2 switch conversion. LNCAROD in cytoplasm competitively binds to miR-145-5p and then upregulates PKM2 to promote aerobic glycolysis	(56)
LncAY	Up	YTHDF2	Promotes HCC proliferation and migration	YTHDF2 increases the half-life of LncCY, promotes the expression of BMI1, and activates the Wnt/β-catenin signaling pathway	(57)
GATA3-AS	Up	KIAA1429	Promotes HCC proliferation and metabolism	KIAA1429 induces m6A methylation at the 3 ‘end of GATA3 pre-mRNA, leading to the separation of HuR and degradation of GATA3 pre-mRNA. GATA3-AS preferentially interacts with KIAA1429 and GATA3 pre-mRNA, inhibiting the expression of GATA3	(58, 59)
ILF3-AS1	Up	METTL3 IGF2BP1	Promotes HCC cell proliferation, migration and invasion	ILF3-AS1 increases the stability of ILF3 mRNA through m6A methylation mediated by METTL3-IGF2BP1	(60)
DUXAP8	Up	METTL3	Promotes HCC progression and stem cell properties	METTL3 promotes DUXAP8 stability and competitively binds miR-584-5p to activate the target gene MAPK1 and the MAPK-ERK signaling pathway	(61)
LINC00106	Up	METTL3 IGF2BP1	Enhances the stemness and metastasis of HCC cells	METTL13/IGF2BP1-mediated m6A modification promotes the expression of LINC00106, competitively binds to sponge let7f, and affects downstream periostin mRNA and the PI3K-Akt signaling pathway	(62)
LINC01273	Up	METTL3 YTHDF2	Promotes HCC sorafenib resistance	LINC01273 promotes the stability of miR-600, enhances the inhibitory effect of miR-600 on METTL3 mRNA, leads to the downregulation of METTL3 expression, and promotes HCC cell sorafenib resistance. LINC01273 can be recognized and degraded by METTL3/YTHDF2.	(63)
NIFK-AS1	Up	METTL3 IGF2BP1	Promotes malignant HCC characteristics; increases sorafenib resistance	The METTL3/IGF2BP1 axis can improve the stability of NIFK-AS1 RNA, increase the expression of NIFK-AS1, and affect miR-637/AKT1/MMP-7 and MMP-9 axis. The uptake and transport of sorafenib by transporter OATP1B1/OATP1B3 are reduced.	(64)

### 4.1 Promotion of Lipogenesis

LncRNAs are involved in many processes of liver fat metabolism, including lipolysis, fatty acid β oxidation and fat differentiation, leading to the occurrence of diseases. During HCC, organisms acquire nutrients through reprogramming and metabolic pathways that affect the metabolism of glucose, fatty acids and amino acids to satisfy the growth and development of cancer cells (21).

It has been reported that the lncRNA and m6A methylation recognition protein “reader” IGF2BP2 plays a role in the proliferation and metabolism of colorectal cancer. LINRIS inhibits ubiquitination and enhances the stability of IGF2BP2 by binding to K139 of IGF2BP2. IGF2BP2 binds to the m6A site of Myc mRNA, enhancing Myc axis-mediated glycolysis and promoting colorectal cancer cell proliferation (65). However, the mechanism of lncRNA and m6A regulatory factors in HCC is different from that in colorectal cancer (31). m6A methylation affects the stability of lncRNAs, and lncRNAs competitively bind miRNAs through a ceRNA-dependent mechanism to promote fat generation and metabolism. METTL3 methylase regulates the downregulation of lncRNA-MEG3, which acts as a molecular sponge to promote liver lipogenesis and metabolism by competitively binding miR-21 and LRP6 (53). METTLL3-dependent m6A modification increases the stability of LINC00958, inhibits miR-3619-5p expression, increases HDGF expression and promotes lipogenesis (55) (**Figure 3A**).

These results suggest that m6A modification may be closely related to lipid metabolism in metabolic reprogramming, which provides the possibility to search for tumor metabolic targets. However, there are few studies on the role of m6A modification in tumor metabolic reprogramming (eg. fatty acid and amino acid metabolism), so more regulatory details need to be explored in this area.

### 4.2 Effects on HCC Processes

The interaction between m6A modification and lncRNA involves two aspects. M6A modification affects RNA–protein interactions, participates in lncRNA-mediated ceRNA models, and participates in lncRNA-miRNA interactions. In addition, lncRNAs can also regulate the expression and function of m6A modification by regulating the m6A modification factor (**Figure 3**).

MEG3 is expressed at low levels in HCC, and m6A methylation occurs in the presence of METTL3. Through the ceRNA mechanism, MEG3 competitively promotes the expression of miR-544b, downregulates the target gene BTG2 and inhibits the proliferation of HCC (54). LNCAROD is highly expressed in HCC patients with a poor prognosis. Increased m6A methylation of METTL3-IGF2BP1 mediates the stability of LNCAROD, thus maintaining its high expression in HCC. In the nucleus, LNCAROD directly binds with serine and arginine-rich splicing factor 3 (SRSF3) to induce the conversion of PKM1 to PKM2. However, LNCAROD in the cytoplasm acts as the molecular sponge of miRNA. The inhibitory effect of miR-145-5p on PKM2 levels was alleviated through the ceRNA mechanism. The expression of hypoxia-inducing factor 1α (HIF1α), which can be triggered by hypoxia, enhances the PKM2-mediated glycolysis process and further promotes the migration and invasion of HCC cells (56). YTHDF2 is expressed at low levels in HCC, and m6A methylation mediated by YTHDF2 can improve the half-life of lncCY, further promoting the stability of the downstream gene BMI1 mRNA and activating the Wnt-β-catenin signaling axis to affect the proliferation, migration and invasion of HCC (57) (**Figure 3B**).

KIAA1429 is highly expressed in HCC and is associated with prognosis. KIAA1429 induces m6A methylation at the 3’ end of GATA3 premRNA, leading to the separation of HuR and degradation of GATA3 premRNA. As a cis-acting element, lncRNA GATA3-AS preferentially interacts with KIAA1429 and GATA3 premRNA, inhibits the expression of GATA3 and leads to the proliferation and metabolism of HCC (58, 59) (**Figure 2A**). ILF3-AS1 is highly expressed in HCC and can be used as a regulatory factor to recruit METTL3 and form the ILF3-AS/METTL3/ILF3 complex, thereby improving the m6A methylation level and stability of ILF3 mRNA. ALKBH5, as a demethylase, is downregulated in HCC and inhibited by ILF3-AS1, which reduces the demethylation process of ILF3 mRNA. ILF3-AS1 enhances the stability of ILF3 mRNA through m6A methylation mediated by the METTL3-IGF2BP1 axis and promotes cell proliferation, migration and invasion, leading to the occurrence of HCC and further affecting the prognosis of patients (60) (**Figure 2B**).

### 4.3 Enhancement of Tumor Stemness

Tumor stemness plays an important role in cancer progression and has been one of the hot topics in cancer research in recent years. It is an important cause of chemoradiotherapy tolerance, tumorigenesis, metastasis, self-replication, metabolic reprogramming and changes in the immune microenvironment (66, 67). However, the exact mechanism is unclear. Studies have shown that lncRNAs play an important role in the regulation of hepatocellular carcinoma stemness.

Abnormal expression of lncRNAs and m6A methylases has been reported to affect tumor cell stemness in pancreatic cancer and breast cancer (68, 69). However, there are few reports that abnormal m6A regulatory factors of lncRNAs affect the stemness of HCC cells.LncRNA DUXAP8 increases the m6A level through METTL3 and its own stability and activates downstream target genes MAPK1 and MAPK-ERK signaling pathways through the competitive binding of miR-584-5p to promote the process and stem cell characteristics of HCC (61). METTL3/IGF2BP1-mediated m6A modification promotes LINC00106 expression. Then, increased LINC00106 competitively binds to let7f through a ceRNA mechanism, affects downstream periostin mRNA and the PI3K-AKT signaling pathway and enhances stemness and metastasis of HCC cells (62) (**Figure 3C**). This evidence suggests that m6A can control the expression and function of lncRNAs, thus regulating tumor stemness. It is worth pointing out that lncRNA and m6A modification molecules have potential clinical value in regulating tumor immunity and immune invasion patterns, have potential as tumor stemness markers and therapeutic targets in many cancer species and contribute to improving diagnostic accuracy. At the same time, the study of the drug resistance mechanism of tumor stem cells has good application prospects and significance, which is of great significance to the adjuvant drug therapy of HCC patients.

### 4.4 Influence of Drug Resistance on Tumor Cells

Drug resistance of tumor cells to chemotherapeutic drugs is one of the main reasons for failure of tumor chemotherapy. After the tumor is resistant to chemotherapy, it easily progresses to tumor recurrence and metastasis, so 80% ~ 90% of the deaths of tumor patients can be directly or indirectly attributed to drug resistance (59). The generation of drug resistance is a complex process caused by a variety of factors, among which abnormal methylation of lncRNAs and m6A can also lead to drug resistance in HCC patients (70).It has been found that lncRNA-TSUC7 modified by the m6A methylated binding protein YTHDF2 through the Notch signaling pathway affects erlotinib resistance in lung cancer (71). METTL3 promotes the stability of lncRNA-TRIM11 transcripts through the m6A-IGF2BP2-dependent pathway and enhances cisplatin resistance in nasopharyngeal carcinoma (72). In HCC, abnormal m6A methylation modification of lncRNAs mainly causes sorafenib resistance.

LINC01273 is highly expressed in HCC and sorafenib-resistant patients and affects tumor drug resistance through a dual feedback mechanism. On the one hand, there were m6A methylation modification sites on LINC01273, which were recognized by METTL3/YTHDF2 and degraded, leading to the combination of miR-600 and METTL3 mRNA, and the high expression of METTL3 promoted oncogene and resistance to sorafenib. On the other hand, LINC01273 colocalizes with miR-600 in the HCC cell cytoplasm and acts as a molecular sponge of miRNA, which increases the stability of miR-600 and promotes the inhibition of miR-600 on METTL3 mRNA, leading to the downregulation of METTL3 expression. HCC cells undergo autophagy, which affects resistance to sorafenib (63). NIFK-AS1 expression was upregulated in HCC cells and tissues. The stability of NIFK-AS1 RNA was enhanced in the process of m6A methylation mediated by the METTL3/IGF2BP1 axis, which promoted the high expression of NIFK-AS1. Through the ceRNA mechanism, the miR-637/AKT/MMP-7/MMP-9 axis is affected to drive the malignant characteristics of HCC. The high expression of NIFK-AS1 reduced the uptake and transport of the transporter OATP1B1/OATP1B3 to sorafenib and enhanced the resistance of HCC patients to sorafenib (64) (**Figure 3D**).

## 5 Discussion

In summary, m6A regulatory factors (including “Writer”, “Reader” and “Eraser”) and lncRNAs are closely related to HCC processes, including tumor cell proliferation, metastasis, cancer cell stemness and tumor drug resistance. Currently, the m6A methylation modification of lncRNAs in HCC mainly focuses on METTL3 and IGF2BP1/2/3 m6A regulatory factors. LncRNAs modified by m6A methylase contain methylation sites, which can affect their stability and expression. MiRNA competitively binds lncRNAs through the ceRNA mechanism to modulate the expression of downstream target molecules and promote the occurrence of HCC. Some antisense lncRNAs, which serve as regulatory factors, affect the binding of m6A methylase to mRNA, which has an impact on the pathogenesis of HCC. However, how other types of lncRNAs play a role in the HCC process has not been reported. How m6A demethylation participates in lncRNA expression has not been reported, partly due to the controversy over the expression level of FTO in HCC, and only a few kinds of demethylases have been described. Whether there are other types of demethylases remains to be further explored. Currently, some m6A inhibitors (such as FTO class inhibitors) have shown important functions in tumors. They alter the overall level of m6As in tumor cells by targeting the inhibition of m6A regulatory factors, providing new therapies for tumors. New methods and technologies need to be developed so that the dynamic modification process of m6A regulatory factors and the influence of m6A methylation modification on lncRNA subcellular localization can be investigated, along with the underlying mechanism of action. It is crucial to reveal how epigenetic transcriptomic modification affects the occurrence of diseases in lncRNAs.

At the same time, changes in lncRNA and m6A levels under hypoxia can activate aerobic glycolysis in tumor cells and may alter the intracellular tumor microenvironment or cellular metabolism, suggesting that the occurrence of HCC is the result of multiple factors. LncRNA and m6A methylation mutually regulate the occurrence and development mechanism of HCC, and such multidirectional regulation may help clinicians and researchers gain a better understanding of HCC.

LncRNAs and m6A regulatory factors play significant roles in a variety of biological processes of cells, and their abnormal expression is associated with the diagnosis and prognosis of a variety of diseases, including cancer. LncRNAs and m6A-regulatory factors can be used as specific molecular markers or prognostic markers for HCC. The combination of lncRNAs and m6A regulatory factors can be more sensitive and accurate for the detection of HCC, which can help improve the prognosis of patients and reduce the mortality of patients. Both lncRNA and m6A methylated modified protein are expected to be tumor markers, but lncRNA is far less unstable than m6A methylase. m6A methylation can regulate the stability of lncRNA and improve the effectiveness and accuracy of tumor markers by adding labels to lncRNA. Some lncRNAs are expressed differently in serum. Studies have shown that the combination of the two growth factors in serum provides a new auxiliary method for the diagnosis of HCC, and its combination with AFP has a higher predictive value for HCC (73, 74),the clinical application of AFP and other m6A regulatory proteins can be used for the early detection and diagnosis of HCC. It has been reported that the therapeutic efficacy of PD-1 checkpoint blockade is diminished in METTL3 deficient mice, thus identifying Mettl3 as a potential therapeutic target for tumor immunotherapy, targeted intervention of lncRNA can affect the signaling pathway of immune cells to improve the chemotherapy sensitivity of tumors (75). The development of siRNA(lncRNA)-NPS nanomaterials based on PLGA-PEG can increase the uptake of drugs by tumor cells, reduce the biological function of tumor cells, and significantly improve the tumor targeting ability in the body, which can be used as an important target for clinical treatment of cancer patients (55). In-depth understanding and monitoring of the progression of cancer is a crucial link in cancer treatment, which can provide a theoretical basis for new targeted therapy and immunotherapy and design individualized treatment plans for the benefit of liver cancer patients.

Although two newly developed high-throughput deep sequencing technologies (MeRIP and m6A sequencing) play a key role in revealing the functional significance of m6A modification, there are still many unknown lncRNAs to be excavated due to the variety and complexity of lncRNAs, and multiple m6A methylation modification sites on lncRNAs. The effect of single base modification sites or multiple base modification sites interaction on lncRNAs remains to be studied. At present, most studies are devoted to elucidating the downstream mechanism of differential expression of lncRNAs in tumors, while its upstream regulatory mechanism has not attracted attention. m6A inhibitors or activators interfere with lncRNA expression levels, and new methods to regulate lncRNAs with different expressions are yet to be explored. The current development of the possible combination of lncRNAs and m6A as potential targets for tumor diagnosis and treatment is mostly confined to mechanism studies, and further validation is needed for clinical application.

## Author Contributions

WJZ, WLW, and QM contributed equally to this work. LY, JZY, and ZLT draw the figures. XZ and JLW designed the subject. KST revised the article. All authors contributed to the article and approved the submitted version.

## Funding

This study was supported by the Major Military Logistics Research Projects (AKJ19J001), Xijing Hospital Disciplinary Boost program (XJZT19MJ09), Key Research and Development program of Shaanxi province (2020SF-066) and the National Natural Science Foundation of China (81970566, 82170667,82070681, 81900571).

## Conflict of Interest

The authors declare that the research was conducted in the absence of any commercial or financial relationships that could be construed as a potential conflict of interest.

## Publisher’s Note

All claims expressed in this article are solely those of the authors and do not necessarily represent those of their affiliated organizations, or those of the publisher, the editors and the reviewers. Any product that may be evaluated in this article, or claim that may be made by its manufacturer, is not guaranteed or endorsed by the publisher.
